# HOXD-AS1 promotes the epithelial to mesenchymal transition of ovarian cancer cells by regulating miR-186-5p and PIK3R3

**DOI:** 10.1186/s13046-019-1103-5

**Published:** 2019-03-01

**Authors:** Shanshan Dong, Ranran Wang, Hui Wang, Qi Ding, Xiao Zhou, Jing Wang, Keqiang Zhang, Ying Long, Shan Lu, Ting Hong, Huayi Ren, Kee Wong, Xiaowu Sheng, Yu Wang, Yong Zeng

**Affiliations:** 1 Translational Medicine Center, The Affiliated Cancer Hospital of Xiangya School of Medicine, Central South University/Hunan Cancer Hospital, Changsha, China; 2 Engineering Technology Research Center for diagnosis-treatment and application of tumor liquid biopsy, Changsha, China; 3 Key Laboratory of Radiation Oncology, Department of Radiation Oncology, Hunan Cancer Hospital and The Affiliated Cancer Hospital of Xiangya School of Medicine, Central South University, Changsha, Hunan China; 4 The fifth department of gynecological oncology The Affiliated Cancer Hospital of Xiangya School of Medicine, Central South University/Hunan Cancer Hospital, Changsha, China; 5 Department of Biochemistry and Molecular Biology, Second Military Medical University, Changsha, China; 6 Division of Cellular and Molecular Research, National Cancer Centre Singapore, Singapore, Singapore

**Keywords:** HOXD-AS1, EMT, miR-186-5p, PIK3R3, lncRNA, ceRNA, EOC

## Abstract

**Background:**

Epithelial ovarian cancer (EOC) is one of the most malignant gynecological tumors worldwide. Deregulation of long non-coding RNAs (lncRNAs) has been implicated in various oncogenic processes in multiple cancers. In this study, we aim to identify and characterize clinically relevant lncRNA deregulation in EOC.

**Methods:**

LncRNAs, mRNAs and miRNAs were profiled using expression microarrays and validated using reverse transcription quantitative PCR in EOC cells and tissues. siRNAs targeting either HOXD-AS1 or PIK3R3 together with miR-186-5p inhibitors were used to modulate endogenous target expression in EOC cell lines in vitro. In vitro wound healing assay, trans-well assay, Western-blot assay,and Dual-luciferase reporter assay were used to explore the biological roles and molecular function underlying HOXD-AS1 in the EOC cells. Progression-free survival (PFS) and overall survival (OS) were statistically analyzed by Kaplan-Meier method test.

**Results:**

HOXD-AS1 was found to be significantly over-expressed in EOC tumors. High HOXD-AS1 expression significantly correlated with poorer PFS and OS of EOC patients. Multivariate Cox proportional hazards modeling indicated that HOXD-AS1 was an independent risk predictor of EOC patients (HR = 1.92, *p* = 0.004). SiRNA inhibition of HOXD-AS1 reduced cell migration, invasion, and epithelial-mesenchymal transition (EMT) in EOC cells in vitro by preventing HOXD-AS1 directly binding to miR-186-5p, and resulting in down-regulating of PIK3R3. The novel HOXD-AS1/miR-186-5p/PIK3R3 pathway was clinically relevant as we observed a significantly inverse correlation between HOXD-AS1/miR-186-5p and between miR-186-5p/PIK3R3 in an independent cohort of 200 EOC tissues.

**Conclusions:**

HOXD-AS1/miR-186-5p/PIK3R3 is a novel pathway to promote cell migration, invasion, and EMT in EOC.

**Electronic supplementary material:**

The online version of this article (10.1186/s13046-019-1103-5) contains supplementary material, which is available to authorized users.

## Background

EOC is the third most common and second most lethal gynecological malignancy worldwide [1]. The standard treatment is maximal cytoreductive surgery followed by platinum-based chemotherapy regimen [2]. However, the occurrence of metastasis and acquired drug resistance often lead to poor prognosis in patients with advanced ovarian cancer [3, 4]. Therefore, there is an urgent clinical need to understand the molecular mechanisms leading to the occurrence of tumor metastasis and drug resistance in EOC. In addition, novel biomarkers which can accurately predict tumor metastasis and drug resistance are needed to provide valuable insight into the clinical management of EOC.

EMT is a biological process in which polarized epithelial cells adopt mesenchymal cell phenotype which includes enhanced migratory capacity, invasiveness, elevated resistance to apoptosis, and greatly increased production of extracellular matrix components [5, 6]. It has been well demonstrated that the activation of EMT program endows cancer cells with the multiple features that are required to execute most steps of the invasion-metastasis cascade [7, 8]. In addition, activation of EMT pathway induces chemoresistance in multiple cancers such as pancreatic cancer [9], lung cancer [10, 11], gastric cancer [12], liver cancer [13] and EOC [14].

LncRNA and miRNAs both belong to non-protein encoding RNAs which are implicated to play important roles in a variety of cellular processes such as cell proliferation, apoptosis, and migration [15, 16]. The aberrant expression of lncRNAs in tumors can induce EMT and increase the invasion and migration of tumor cells. For example, XIST, which is a lncRNA over-expressed in colorectal cancer tissues and cells, can promote invasion, proliferation, and EMT progress through XIST/miR-200b-3p/ZEB1 pathway [17]. Another example is PVT1, a highly expressed lncRNA in prostate cancer cells that can co-regulate Twist with miR-186 and promote tumor invasion, migration, and the progress of EMT [18]. This lncRNA/miRNA/mRNA pathway has been proposed as a new model by which lncRNAs regulate target gene expression through miRNA response elements [19]. Salmena et al. have termed this phenomenon as the competing endogenous RNA (ceRNA) theory [20].

In this study, we identified a HOXD-AS1/miR-186-5p/PIK3R3 pathway in EOC as another example exhibiting the ceRNA property. The HOXD-AS1/miR-186-5p/PIK3R3 pathway plays an important role in invasion, migration and EMT of EOC.

## Methods

### Patients and EOC tissue samples

We collected EOC (*n* = 36) and normal ovarian (*n* = 14) tissue samples from patients who underwent surgery at the Hunan Cancer Hospital (Affiliated Cancer Hospital of Xiangya School of Medicine, Central South University, Changsha, Hunan, China) from 2012 to 2014. The resected tissues were separately identified and confirmed by two pathologists and kept in liquid nitrogen immediately for subsequent use. In addition we also collected formalin-fixed paraffin-embedded (FFPE) specimens from an independent cohort of 200 patients to analyze the relationship of PFS and OS with the expression levels of HOXD-AS1. The clinicopathologic characteristics of patients are shown in Table 1. The ethics committee of Hunan Cancer Hospital approved the protocol, and all patients signed informed consents before sample collection.

**Table 1:** Top ten significantly up-regulated and down-regulated lncRNAs in EOC

Top ten up-regulated lncRNAs			
	lncRNA ID	FC	*p*
1	CTD-2116 N17.1	54	0.001
2	MAL2	50	0.006
3	LINC00707	19	0.005
4	CXADRP3	18	0.001
5	HOXD-AS1	18	0.007
6	CDKN2B-AS1	16	0.004
7	ALDH3B1	15	0.000
8	XXbac-BPG254F23.6	11	0.004
9	HCP5	10	0.004
10	LINC00152	9	0.000
Top ten down-regulated lncRNAs			
	lncRNA ID	FC	*p*
1	LINC00473	− 132	0.000
2	PWRN1	− 85	0.004
3	TRHDE-AS1	−38	0.000
4	ADAMTS9-AS2	−33	0.000
5	LINC00478	− 28	0.002
6	CECR7	−28	0.000
7	CTC-454 M9.1	−26	0.000
8	ADAMTS9-AS1	−26	0.007
9	NR2F2-AS1	− 22	0.006
10	AL132709.5	−22	0.007

### Differentially expressed gene analysis

Differentially expressed genes and lncRNAs were identified using the threshold of fold change ≥2 and Student’s t-test *p*-value < 0.01 between EOC tumor versus normal tissues. The R package, Significance analysis of microarray (SAM) was used to identify differentially expressed miRNA at a threshold of fold change≥1.5, false discovery rate (FDR) q-value< 0.05. Hierarchical clustering was performed on the pheatmap package of R language and heatmap was constructed. Volcano maps were drawn using the ggplot2 packages.

### Gene ontology (GO) and Kyoto encyclopedia of genes and genomes (KEGG) analysis

DAVID (The Database for Annotation, Visualization and Integrated Discovery, https://david.ncifcrf.gov/) was used to annotate the function of differentially expressed mRNA and enrichment of the signaling pathway. GO included cell component, biological process, and molecular function. Aberrant mRNAs were enriched into different pathways according to the KEGG database. TOP 10 terms were presented. The recommended *p*-value cutoff of *p* < 0.05 was used.

### Target gene prediction

The potential miRNAs that may bind to HOXD-AS1 were predicted by DIANA (http://carolina.imis.athena-innovation.gr/diana_tools/web/index.php?r=lncbasev2%2Findex-predicted). The candidate mRNA that may bind to miR-186-5p were predicted by starBase v2.0 (http://starbase.sysu.edu.cn/) database, which integrates five programs: targetScan, picTar, RNA22, PITA, and miRanda/mirSVR. Candidate mRNA are defined as the genes which are predicted to be target mRNA of miR-186-5p by at least three programs.

### Cell culture

EOC cell line A2780 was cultured in Dulbecco’s modified Eagle’s medium (TransGen, Beijing, China), and EOC cell line SKOV3 and normal ovarian cell line IOSE80 were cultured in RPMI-1640 (TransGen, Beijing, China) with 10% fetal bovine serum (Gibco) and 1% penicillin/streptomycin (TransGen, Beijing, China). All of the cells were cultured in an incubator at 37 °C with 5% CO_2_.

### RNA extraction and RT-qPCR

Total RNAs from tissues and cells were extracted using Trizol reagent (Invitrogen). Total RNAs from FFPE were extracted using RNeasy®FFPE Kit (Qiagen, Germany). cDNA was synthesized with the TransScript® II One-Step gDNA Removal and cDNA Synthesis SuperMix Kit (TransGen, Beijing, China) according to the manufacturer’s instructions. RT-qPCR was performed on the LightCycler®96 system (Roche) using a standard protocol from the TransStart® Tip Green qPCRSuperMix Kit (TransGen, Beijing, China). The results were normalized to GAPDH. The sequences of primers used for RT-qPCR assays are listed in Additional file 1.

For the detection of miRNA expression, reverse transcription and qPCR were performed using stem-loop primers and Bulge-Loop™ miRNA RT-qPCR Starter Kit from Ribobio (Guangzhou, China). U6 snoRNA was used as the endogenous control. All experiments were performed in triplicate according to the manufacturer’s manual. Expression fold changes were calculated using 2^-∆∆Ct^ methods.

### In situ hybridization (ISH)

In situ hybridization was performed to detect the expression of HOXD-AS1, miR-186-5p, and PIK3R3. The probes of HOXD-AS1, miR-186-5p, and PIK3R3 were designed by BOSTER (Wuhan, China). RNA ISH was performed using the in situ hybridization detection kit (BOSTER) and DAB chromogenic kit (ZSGB-BIO) on 3-μm FFPE tissue sections according to the manufacturer’s instructions. The staining scores were determined based on both the intensity and proportion of target gene-positive cells under 20X and 40X objective.

### Small interfering RNA (siRNA) and miRNA inhibitor transfections

LncRNA HOXD-AS1, siRNA (si-HOXD-AS1), PIK3R3 siRNA (si-PIK3R3), miR-186-5p inhibitor, and negative control (si-NC, miR-NC) were all obtained from Ribobio (Guangzhou, China). RiboFECT™ CP transfection Kit (Guangzhou, China) was used for cell transfection based on manufacturer’s instructions. SKOV3 and A2780 were seeded in a six-well plate and transfection would be performed when cell’s confluence reached 50%. Cells were transfected with a final concentration of 100 nM of individual siRNA and 200 nM of miR-186-5p inhibitor. All of the steps were carried out according to the manufacturer’s specification. Experiments were performed three times.

### Wound healing assay

The cell monolayer was scraped in straight lines using a 10ul pipette tip and washed with PBS two times when transfected cells were up to 80% confluence in a six-well culture dish. The cells were cultured in culture medium with 3% FBS and 1% penicillin/streptomycin (TransGen, Beijing, China). Images were captured at 0, 12, 24, 36, and 48 h following the initial scratch from five different areas for each wound. We used Image J (National Institutes of Health, Bethesda, MD, USA) software to calculate cell wound healing rate as: (the original wound areas - the actual wound areas at different times)/ (the original wound areas).

### Trans-well assays

The invasion ability was measured using transwell chambers coated with 1:30 diluted Matrigel Basement Membrane Matrix (Corning Life Sciences, NY, USA). Migration assays were performed without matrigel. 1 × 10^5^ cells were seeded into upper chambers (8 μM pore size, Corning Life Sciences, NY, USA) with 200ul serum-free media. A total of 600 ul of 20% FBS media was added into the lower chambers. After incubation for 48 h at 37 °C, the cells in the upper chamber that did not invade through the pores were wiped out with cotton wool. The cells in the lower chamber were fixed with methanol and stained with 0.5% crystal violet. Cells were counted and imaged at × 200 magnification. The experiments were repeated three separate times.

### Western blotting

After transfection for 48 h, cells were lysed using RIPA buffer (Thermo, USA) with proteinase inhibitor cocktail (Roche), then incubated at 4 °C for 15 min. The lysate was centrifuged and protein concentration was measured by BCA Protein Assay Kit (Thermo, USA). The protein was separated on 10% sodium dodecyl sulfate- polyacrylamide gel electrophoresis (SDS-PAGE) and then transferred to 0.22 μm PVDF membrane (Thermo, USA). After blocking with 5% fat-free milk for 1 h, the membranes were incubated with primary antibodies at 4 °C overnight with gentle shaking. Next, the membranes were incubated with secondary antibody at room temperature for 2 h. Finally, the immunofluorescence of protein bands was visualized using Pierce**™** ECL Western Blotting Substrate (32,109, Thermo Scientific, USA) by Image J 2 software (Madison, WI, USA). Primary antibodies are as following: E-cadherin (1: 500, ab15148, Abcam company), Vimentin (1:500, #7431, Cell Signaling Technology (CST)), GAPDH (1:5000, 60,004-l-lg, Proteintech company), Rac1 (1:500, ab33186, Abcam), Twist (1500, GTX60776, Gene Tex).

### Dual-luciferase reporter assay

The dual luciferase reporter plasmids (LncRNA-HOXD-AS1-WT, LncRNA-HOXD-AS1-Mut, PIK3R3-WT, PIK3R3-Mut, miR-186-5p, and negative control) were synthesized by GenePharma (Shanghai, China). The SKOV3 cells were seeded into 96-well plates and cultured to 80% confluence. The plasmid vectors or empty plasmid were transiently transfected into SKOV3 cells using lipofectamine 3000 (Invitrogen). After 48 h incubation, the luciferase activity was examined by Dual-Glo® Luciferase Assay System (Promega, USA) according to the manufacturer’s protocol.

### Statistical analysis

All experiments were performed three separate times. Statistical analysis was performed with SPSS 13.0 and graphs were constructed with GraphPad Prism 5 (La Jolla, CA, USA). The data were presented as the mean ± Standard Deviation (SD). The significance of differences between groups was assessed by Student’s t-test and Chi-square test. The Kaplan-Meier method test was used for PFS analysis and OS analysis. Pearson correlation analysis was used to determine the relationship between two genes. We used the univariate and multivariate Cox proportional hazards modeling to determine the effects of variables on survival. A *p*-value of < 0.05 was considered statistically significant.

## Results

### Transcriptome landscape of EOC

**Fig. 1:**
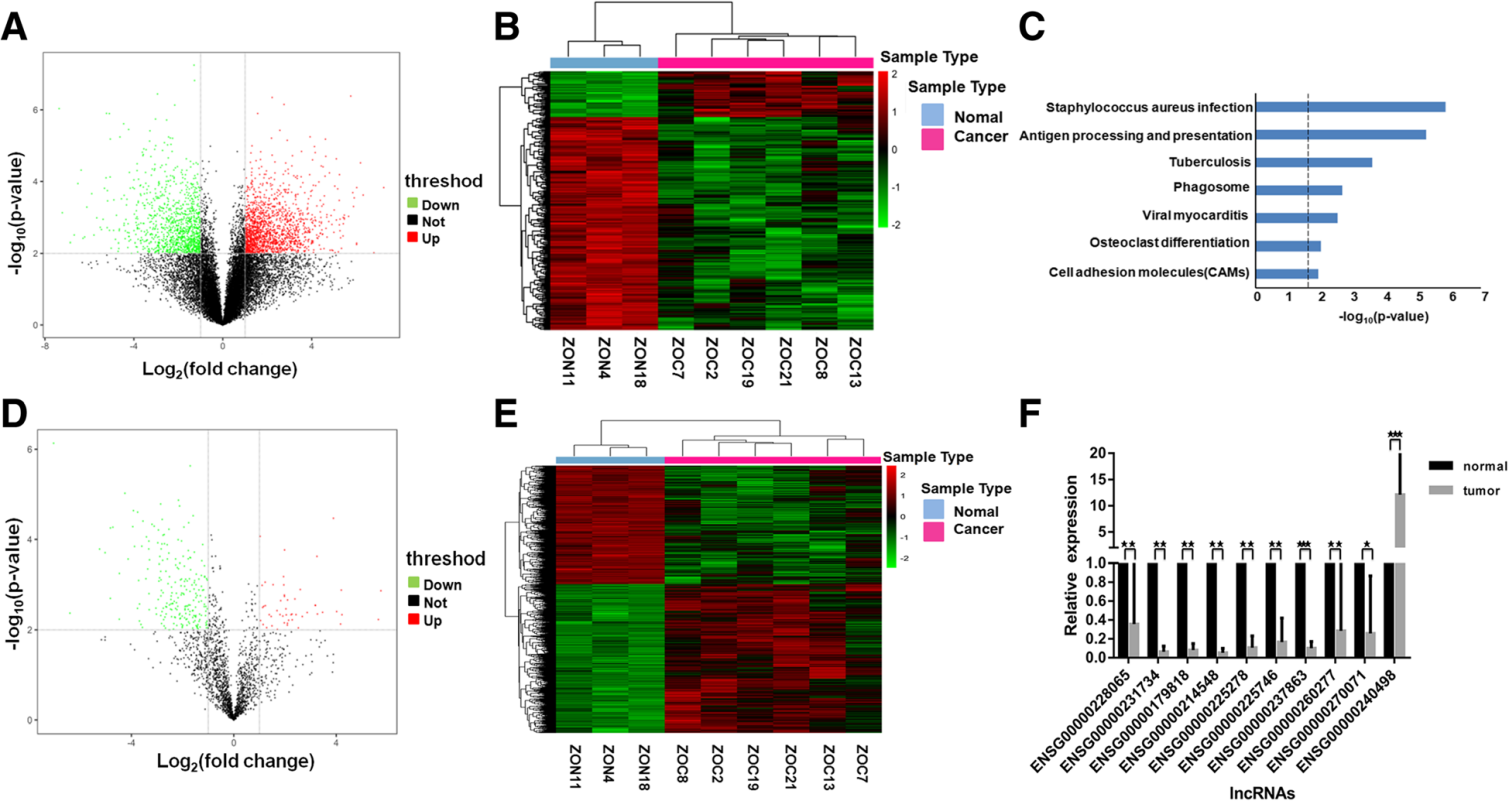
Transcriptomic landscape of epithelial ovarian cancer. **a** Volcano plot showing significantly differentially expressed coding gene mRNAs in six EOC tissues versus three matched normal ovary tissues. 2552 significantly differentially mRNAs were indicated in the two upper lateral quadrants with absolute fold change ≥2 and *p* value < 0.01. **b** Heatmap of the 2552 significantly differentially expressed mRNAs showing clear hierarchical clustering using Pearson correlation and average linkage. **c** KEGG pathway enrichment analysis showing the 2552-gene signature to be significantly enriched in important cellular pathways. **d** Volcano plot showing significantly differentially expressed long non-coding RNAs in six EOC tissues versus three matched normal ovary tissues. 288 significantly differentially lncRNAs were indicated in the two upper lateral quadrants with absolute fold change ≥2 and p value < 0.01. **e** Heatmap of the 288 significantly differentially expressed lncRNAs showing clear hierarchical clustering using Pearson correlation and average linkage. **f** Ten out of the 288 significantly differentially expressed lncRNAs were randomly selected and validated in an independent cohort of 50 patient samples. * indicated statistically significantly differentially expressed lncRNAs in the validation cohort. * denotes *p* < 0.05, **denotes *p* < 0.01, ***denotes *p* < 0.001

We performed transcriptome profiling on 6 human epithelial ovarian cancer tissues and three matched normal ovary samples using the Agilent Human lncRNArray + mRNA Array V4.0. at a threshold of absolute fold change of greater than 2 and p-value of less than 0.01. We observed a total of 2552 mRNAs in EOC to be differentially expressed compared to normal ovary samples, of which 1496 were up-regulated, and 1056 were down-regulated (Fig. 1a and b). These differentially expressed mRNAs were enriched in a number of KEGG pathways such as Straphylococcus aureus infection and cell adhesion molecules which are relevant pathways linking EOC for either increased risk or aggressiveness of the cancer (Fig. 1c). In the 1922 well-annotated lncRNAs on the array, we observed 288 lncRNAs to be differentially expressed (Fig. 1d). Among which, 51 were significantly up-regulated and 237 were significantly down-regulated in EOC tumors versus the normal ovary tissues. These 288 lncRNAs can stratify the samples into distinct clusters based on expression values (Fig. 1e). We validated the robustness of the array data by randomly choosing ten lncRNAs from these 288 differentially expressed lncRNAs in an independent cohort of 50 samples comprising of 36 EOC tissues and 14 normal ovary tissues. All ten lncRNAs showed differential expression between EOC and normal ovary tissues which was in concordance with what we observed in the microarrays. In conclusion, the transcriptome profile constructed from high quality data using the Agilent Human lncRNArray+mRNA Array were reproducible in an independent cohort. Table 1 summarized the top ten most up-regulated and down-regulated lncRNAs in EOC tumors and we selected HOXD-AS1 for further characterization.

### HOXD-AS1 is highly expressed in EOC tissues which negatively impacts patients survival

**Fig. 2:**
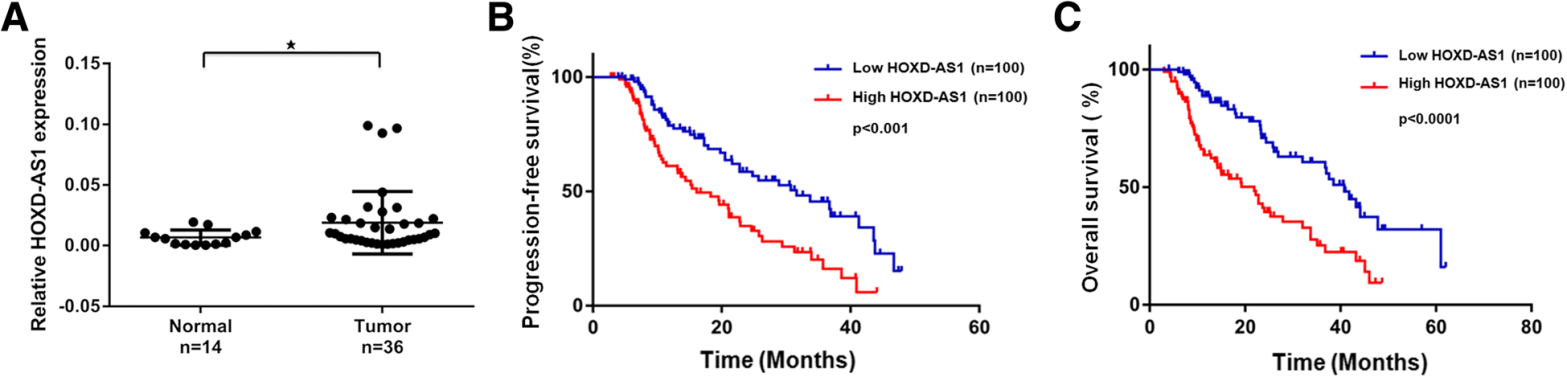
HOXD-AS1 is up-regulated in EOC tissues and predicts poor prognosis. a Relative expression of HOXD-AS1 measured using RT-qPCR and normalized against GADPH in EOC tissues (*n* = 36) and normal ovarian tissues (*n* = 14). Data were expressed as means ± SD. Kaplan-Meier survival analysis showing high HOXD-AS1 expression (above median expression) was significantly associated with poorer progression-free survival (**b**) and overall survival (**c**) of EOC patients (*n* = 200). ^★^ denotes *p* < 0.05, ^★★^denotes *p* < 0.01

We performed RT-qPCR to validate HOXD-AS1 up-regulation in an independent cohort comprising of 36 EOC tissues and 14 normal ovarian tissues and found HOXD-AS1 to be consistently and significantly up-regulated in EOC tissues (*p* < 0.05, Fig. 2a). We further examined HOXD-AS1 expression in another independent cohort of 200 EOC FFPE samples and correlated HOXD-AS1 expression with the clinicopathological features of EOC patients. As shown in Table 2, high HOXD-AS1 expression (above the median value = 0.000565) was positively associated more aggressive EOC tumors characterized by higher tumor grade (Grade G2/3), advanced stage (FIGO stage III/IV), and the presence of lymph node metastasis (p < 0.05). We observed that high HOXD-AS1 level was significantly associated with poorer PFS and OS of EOC patients through Kaplan-Meier analysis (p < 0.05, Fig. 2b and c). Univariate Cox regression analysis showed that the poor prognosis was observed in EOC patients with high-grade tumor, FIGO stage III / IV, lymph node metastasis, and high HOXD-AS1 expression. Multivariate Cox regression analysis suggested that tumor grade, FIGO stage, lymph node metastasis status, and HOXD-AS1 expression are independent risk predictors of prognosis for EOC patients (Table 3). Our data suggested that HOXD-AS1 may play an important functional role in EOC tumor.

**Table 2:** Relationship between HOXD-AS1 expression and clinicopathologic of EOC patients

Characteristics	Number of cases	HOXD-AS1		*p* value	
		Low (*n* = 100)	High (*n* = 100)		
Age (years)				0.479	
<50	95	50	45		
≥ 50	105	50	55		
Histological subtype				0.635	
Serous	145	71	74		
Other	55	29	26		
Grade				0.002	**
G1	82	52	30		
G2/G3	118	48	70		
FIGO stage				0.002	**
I/II	46	32	14		
III/IV	154	68	86		
Lymph node metastasis				0.011	*
Negative	133	75	58		
Positive	67	25	42		
Serum CA125				0.064	
<500	113	50	63		
≥ 500	87	50	37		
Ascites				0.396	
Absent	100	53	47		
Present	100	47	53		

*FIGO* International Federation of Gynecology and Obstetrics

**Table 3:** Univariate and multivariate analysis^a^ of clinicopathological parameters in association with overall survival^b^

Variable	Univariable analysis				Multivariate analysis			
	HR	95% CI	*p* value		HR	95% CI	*p* value	
Age	0.82	0.54–1.25	0.352					
Histological subtype	0.82	0.50–1.35	0.44					
Grade	2.02	1.19–3.41	0.009	*	1.88	1.13–3.13	0.015	*
FIGO stage	2.63	1.29–5.34	0.008	*	2.27	1.16–4.46	0.017	*
Lymph node metastasis	1.92	1.19–3.10	0.008	*	1.95	1.20–3.15	0.007	*
Serum CA125	0.98	0.62–1.55	0.925					
Ascites	0.70	0.45–1.08	0.11					
HOXD-AS1 expression	1.94	1.22–3.06	0.005	*	1.92	1.23–3.00	0.004	*

^a^Cox hazard model

^b^200 patients with full clinical information including age, FIGO stage,histological type,type of chemotherapy and type of surgery.HR,hazard ratio;CI,confidence interval;FIGO, International Federation of Gynecology and Obstetrics; CA125, carbohydrate antigen 125; HOXD-AS1, HOXD Cluster Antisense RNA 1

### HOXD-AS1 promotes EOC cell migration, invasion, and EMT transition

**Fig. 3:**
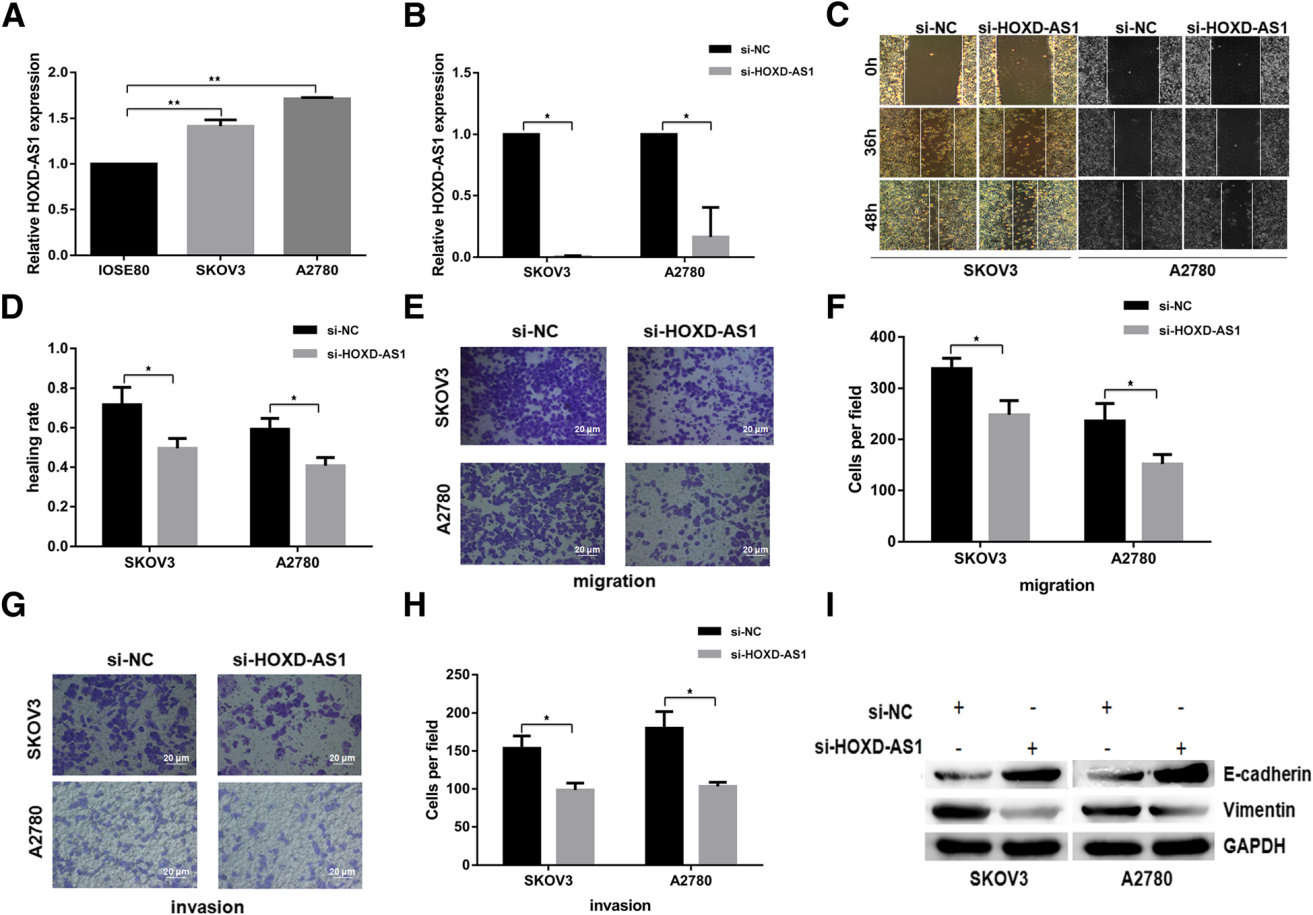
HOXD-AS1 inhibition reduces EOC cell migration, invasion and EMT. **a** HOXD-AS1 was observed to be highly expressed in two EOC cell lines (A2780, SKOV3) compared with normal ovarian cell (IOSE80) using RT-qPCR and normalized to GAPDH as endogenous control. **b** Relative HOXD-AS1 expression in SKOV3 and A2780 cells transfected with siRNA against HOXD-AS1 (si-HOXD-AS1) or negative control (si-NC), measured using RT-qPCR and normalized against GAPDH. **c, d** 2D scratch wound assay showing significantly slower wound healing rate in EOC cells transfected with si-HOXD-AS1 than control cells. **e, f** Trans-well migration assay showing significantly reduced cell mobility through membrane with 8 um pore size in EOC cells transfected with si-HOXD-AS1 than control cells (imaged at × 200 magnification). **g, h** Trans-well Matrigel invasion assay showing significantly reduced ability to invade the membrane with 8 um pore size by EOC cells transfected with si-HOXD-AS1 compared to control cells. The images in (**c**, **e** and **g**) showed a representative experiment while the bar charts in (**d**, **f** and**g**) showed Mean ± SD of quantified data from at least three independent experiments respectively. ^★^ denotes *p* < 0.05, ^★★^denotes *p* < 0.01. **i** Western blot showing an increase in epithelial cell marker, E-cadherin and a reduction in mesenchymal cell marker, Vimentin, in EOC cells transfected with si-HOXD-AS1, compared to the control cells (quantitation graph refer to Additional file 3: Figure S2A/B)

To understand the molecular function of HOXD-AS1 on EOC cells, we designed HOXD-AS1-specific siRNAs to inhibit the endogenous HOXD-AS1 expression in SKOV3 and A2780 EOC cell lines, both of which showed higher HOXD-AS1 expression than normal ovarian cell line IOSE80 (Fig. 3a). SKOV3 and A2780 cells showed significantly reduced ability to migrate in wound healing assays (Fig. 3c and d) when HOXD-AS1 was knocked-down (Fig. 3b). EOC cells in trans-well assays transfected with siRNA against HOXD-AS1 consistently demonstrated significantly reduced capacity to migrate or invade when compared to the mock-transfected control cells (Fig. 3e-h). EMT process is closely related to the invasion and migration of cancer cells therefore we examined the EMT markers between the HOXD-AS1 knocked-down cells and control cells. As shown in Fig. 3i, we observed a significant increase in the expression of epithelial marker E-cadherin and a corresponding decrease in mesenchymal marker vimentin in the HOXD-AS1 knock-down cells as compared to controlls. These results suggested that the down-regulation of HOXD-AS1 inhibited the invasion, migration, and EMT process of EOC cells in vitro.

### HOXD-AS1 binds miR-186-5p to promote cell migration, invasion, and EMT

**Fig. 4:**
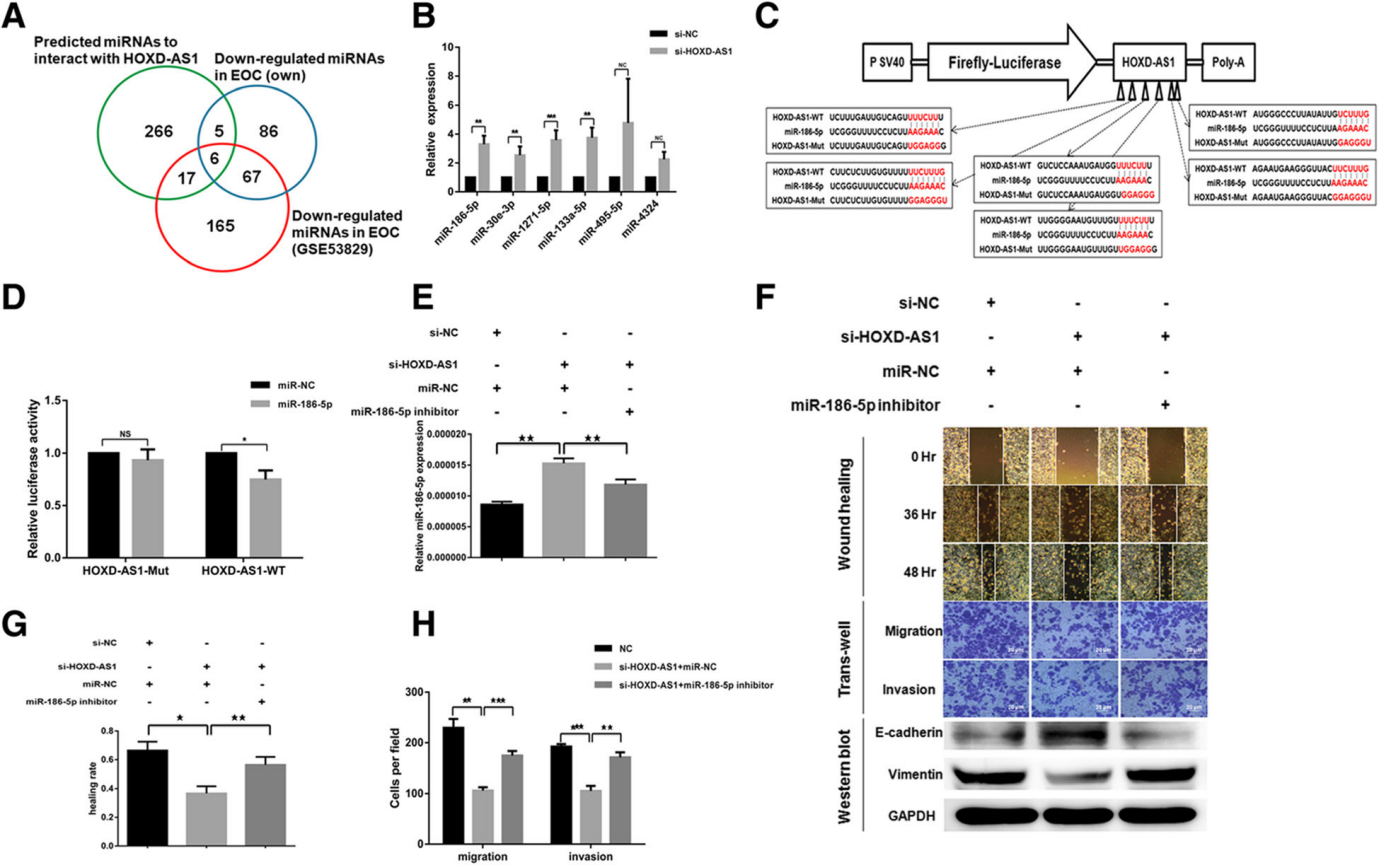
HOXD-AS1 binds to miR-186-5p to promote EOC migration, invasion and EMT. **a** Venn diagram showing the six overlapped miRNAs which were significantly down-regulated in the Affymetrix miRNA arrays and GSE53829, and predicted to be a target of HOXD-AS1. **b** Relative expression of the six miRNAs in (**a**) in EOC cells transfected with siRNA against HOXD-AS1 or si-NC control, measured using RT-qPCR. **c** Schema showing the cloning of wild type as well as a mutant HOXD-AS1 in which all six putative miR-186-5p binding sites were mutated in a luciferase reporter construct. **d** Direct interaction of HOXD-AS1 and miR-186-5p on the putative miR-186-5p binding sites as shown by the specific reduction of relative luciferase reporter activities in cells co-transfected with miR-186-5p and wild type HOXD-AS1 reporter construct. **e** miR-186-5p expression was elevated in EOC cells transfected siRNA against HOXD-AS1 and this could be reversed by the co-transfection of miR-186-5p inhibitors, as shown in RT-qPCR with U6 snoRNA normalization. ^★^ denotes *p* < 0.05, ^★★^denotes *p* < 0.01**. f** EOC cells transfected with si-HOXD-AS1 showed reduced ability to migrate and invade in vitro as measured through wound healing and trans-well assays (imaged at × 200 magnification) than control cells, with a corresponding increase in E-cadherin (an epithelial marker) and decrease in Vimentin (a mesenchymal marker). These changes in phenotypes could be partially rescued with the co-transfection of miR-186-5p inhibitors which partially reversed the miR-186-5p elevation by HOXD-AS1 knock-down, suggesting HOXD-AS1 mediated these phenotypic changes through miR-186-5p (quantitation graph of WB refer to Additional file 3: Figure S2C). **g**, **h** Relative wound healing rate (**g**) and transwell migration/invasion(**h**) from at least three independent experiments as described in(**f**)

We performed Affymetrix miRNA 4.0 chips to evaluate if HOXD-AS1 can increases EOC cell migration, invasion, and EMT through its interaction with miRNAs in a ceRNA manner. We identified a total of 164 miRNAs to be significantly down-regulated in the same nine samples profiled for miRNAs of which 73 were found to be consistently reported by an independent GEO dataset (GSE53829). We further shortlisted a total of 6 miRNAs by overlapping these 73 down-regulated miRNAs in EOC tissues with 293 miRNAs predicted to interact with HOXD-AS1 by DIANA algorithm (Fig. 4a and Table 4). The increased miR-186-5p expression amongst these 6 miRNAs were confirmed upon HOXD-AS1 inhibition (Fig. 4b). We selected miR-186-5p for further research because miR-186-5p consistently showed an inverse correlation with HOXD-AS1 in both patient samples and cell lines. HOXD-AS1 with 6 putative miR-186-5p binding sites further enforced our interest. Inhibition of miR-186-5p promote cell migration, invasion and EMT in EOC cells (Additional file 2: Figure S1).

**Table 4:** List of potentially HOXD-AS1 regulated miRNAs

miRNA ID	miRNA expression in EOC tumors versus normal ovary tissues				Targeting HOXD-AS1 in silico	miRNA changes upon HOXD-AS1 inhibition		
	Affymetrix 4.0		GSE53829		LncBase Score			
	FC	q-value	FC	q-value		FC	*p-*value	
hsa-miR-186-5p	−4.0	0.01	−1.9	0.04	0.93	3.3	0.01	**
hsa-miR-30e-3p	−5.0	0.03	−1.8	0.02	0.77	2.5	0.00	**
hsa-miR-1271-5p	−6.5	0.00	−6.0	0.00	0.76	3.5	0.00	***
hsa-miR-133a-3p	−10.1	0.00	−2.2	0.12	0.99	3.7	0.01	**
hsa-miR-495-3p	−15.7	0.01	−3.2	0.01	0.87	3.6	0.42	NS
hsa-miR-4324	−24.1	0.00	−3.0	0.00	0.87	2.3	0.18	NS

*FC* fold change, q-value, FDR q value; *NS* not statistically significant

To demonstrate that HOXD-AS1 interacts with miR-186-5p through its putative miR-186-5p binding sites, we cloned the wildtype and a mutant HOXD-AS1 in which all six putative miR-186-5p binding sites were mutated and inserted downstream of a firefly luciferase gene (Fig. 4c). As shown in Fig. 4d, we observed significantly reduced reporter activity in the wildtype HOXD-AS1 construct when the cells were co-transfected with miR-186-5p compared to wildtype HOXD-AS1 construct co-transfected with the miRNA controls. However, such difference was abrogated when the putative miR-186-5p binding sites were mutated, indicating that HOXD-AS1 physically interacts with miR-186-5p at its putative binding sites to regulate reporter gene activity. It is further evidenced by concurrent increase in miR-186-5p expression when EOC cells were transfected with siRNAs targeting HOXD-AS1. The HOXD-AS1 knocked-down cells exhibited more epithelial and less mesenchymal phenotype (Fig. 4e, f, g and h, middle panel) which lead to reduced ability to migrate or invade. We observed a corresponding reversal of the above phenotype in cell migration, invasion, and EMT (Fig. 4e and f right panel) when miR-186-5p inhibitors were co-transfected with si-HOXD-AS1 to partly negate the increase in miR-186-5p expression. Therefore, our data demonstrated HOXD-AS1 promotes cell migration, invasion, and EMT through inhibiting miR-186-5p.

### miR-186-5p targets PIK3R3 to negatively regulate cell migration, invasion, and EMT

**Fig. 5:**
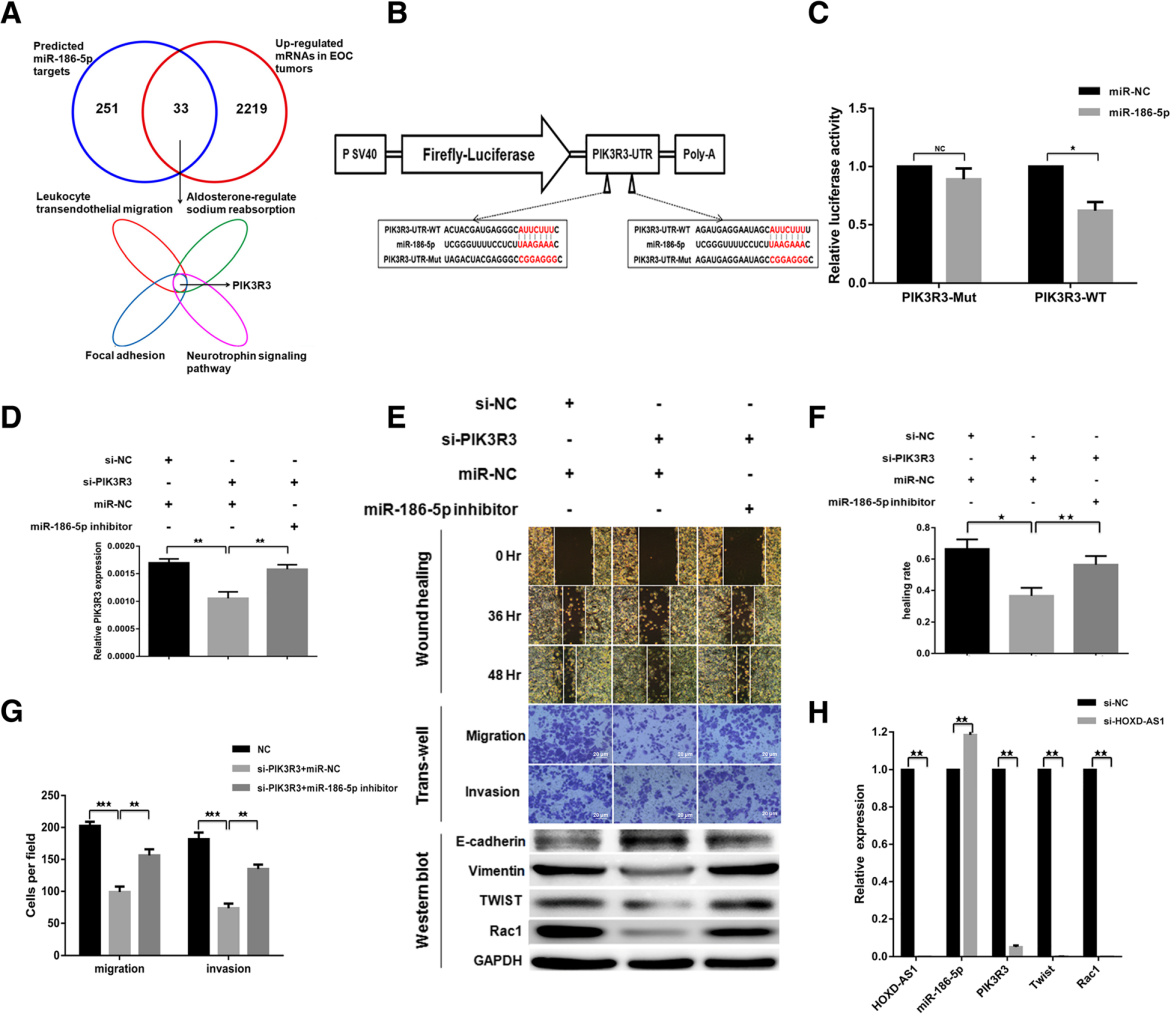
miR-186-5p inhibits migration, invasion and EMT by targeting PIK3R3 in EOC cells. **a** Venn diagram showing the 33 putative miR-186-5p target genes which were significantly up-regulated in the mRNA arrays and predicted to be a target of miR-186-5p. **b** Schema showing the cloning of wild type as well as a mutant PIK3R3 3’UTR in which the two putative miR-186-5p binding sites were mutated in a luciferase reporter construct. **c** Direct interaction of miR-186-5p and PIK3R3 3’UTR on the putative miR-186-5p binding sites as shown by the specific reduction of relative luciferase reporter activities in cells co-transfected with miR-186-5p and wild type PIK3R3 #UTR reporter construct. **d** PIK3R3 expression was suppressed in EOC cells transfected siRNA against PIK3R3 and this could be partly rescued by the co-transfection of miR-186-5p inhibitors, as shown in RT-qPCR with GAPDH normalization. **e** EOC cells transfected with si-PIK3R3 showed reduced ability to migrate and invade in vitro as measured through wound healing and trans-well assays (imaged at × 200 magnification) than control cells, with a corresponding increase in E-cadherin (an epithelial marker) and decrease in Vimentin (a mesenchymal marker) as well as Twist and Rac1 levels. These changes in phenotypes could be partially rescued with the co-transfection of miR-186-5p inhibitors which partially rescued PIK3R3 expression (quantitation graph of WB refer to Additional file 3: Figure S2D/E). **f**, **g** Relative wound healing rate (**f**) and transwell migration/invasion(**g**) from at least three independent experiments as described in(E). **h** Relative expression of HOXD-AS1, miR-186-5p, PIK3R3, Twist and Rac1 in EOC cells transfected with siRNA against HOXD-AS1 or si-NC control

In order to investigate how miRNAs regulate cellular functions through its target genes we queried starBase v2.0 to identify a total of 284 predicted targets of miR-186-5p, among which 33 were significantly up-regulated in EOC tissues with low miR-186-5p expression (Fig. 5a). KEGG pathway enrichment analysis identified four pathways such as focal adhesion all are important for cell migration and invasion. PIK3R3 was involved in all four pathways which suggestes PIK3R3 might be a direct miR-186-5p target. To test this hypothesis, we cloned the wildtype 3′ untranslated region (3’UTR) of PIK3R3 and inserted into the downstream region of the luciferase reporter gene. We mutated the two putative miR-186-5p binding sites along the PIK3R3 3’UTR to construct a mutant clone (Fig. 5b). Specific reduction in luciferase activity was only observed in EOC cells co-transfected with miR-186-5p and wildtype PIK3R3 3’UTR but not the mutant PIK3R3 3’UTR, confirming our hypothesis that miR-186-5p interacts with putative binding sites along PIK3R3 3’UTR to down-regulated luciferase reporter gene expression (Fig. 5c). Furthermore, EOC cells transfected with siRNA against PIK3R3 exhibited greater epithelial phenotypes and reduced ability to migrate and invade. This effect could be rescued by the co-transfection of miR-186-5p inhibitors as our data suggested that miR-186-5p binds to PIK3R3 to negatively regulate cell migration, invasion, and EMT (Fig. 5d, e, f & g). As shown in Fig. 5h, in EOC cells transfected with siRNA against HOXD-AS1, we observed increased miR-186-5p and decreased PIK3R3 along with Twist and Rac1 which contributed to reduced cell migration, invasion and EMT.

### HOXD-AS1/miR-186-5p/PIK3R3 is a clinically relevant pathway regulating EMT in EOC tissues

**Fig. 6:**
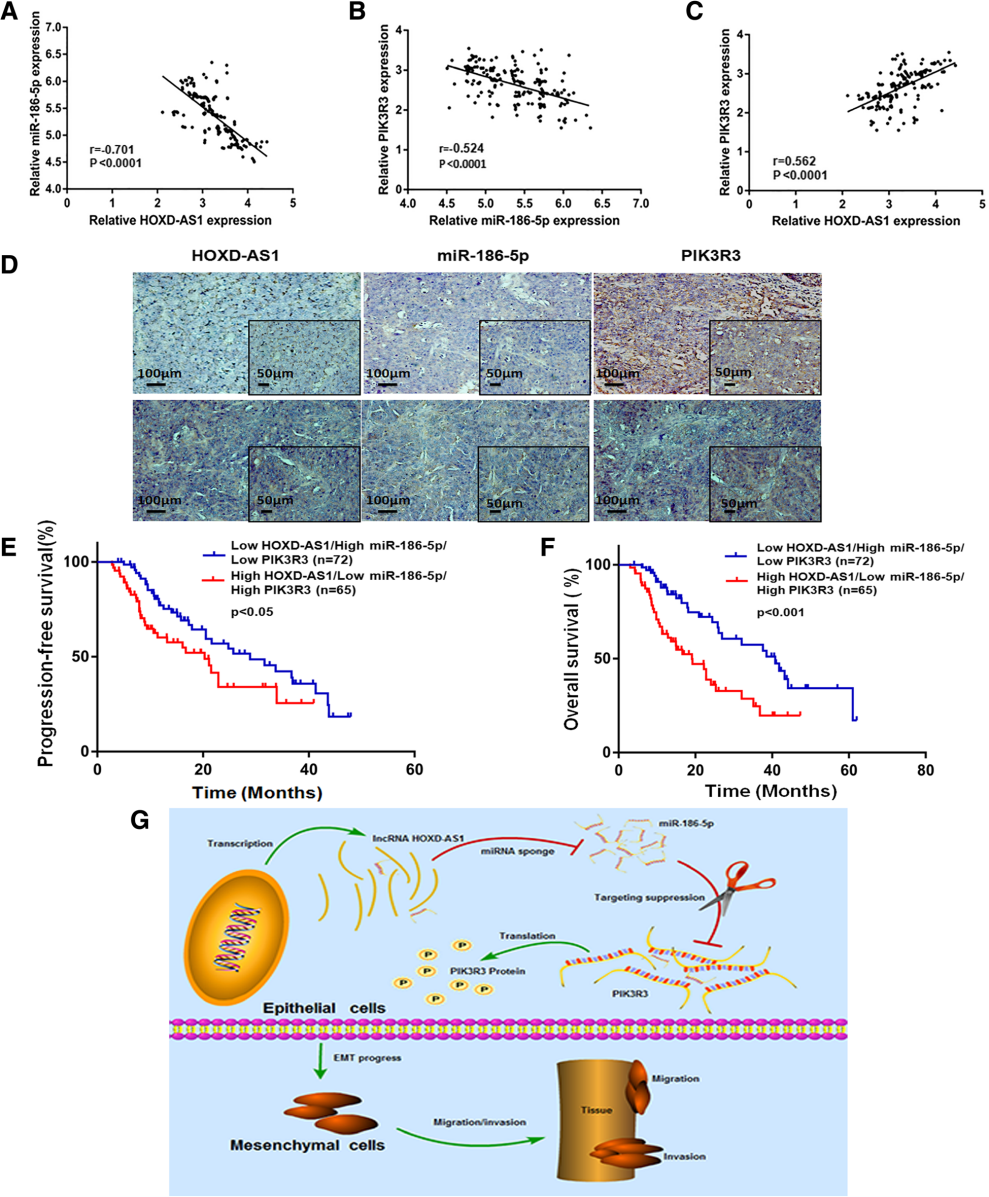
HOXD-AS1/miR-186-5p/PIK3R3 is a clinically relevant pathway. **a** Pearson correlation showing significant inverse correlation between HOXD-AS1 and miR-186-5p in 200 EOC tissues. **b** Pearson correlation showing significant inverse correlation between miR-186-5p and PIK3R3 in 200 EOC tissues. **c** Pearson correlation showing significant positive correlation between HOXD-AS1 and PIK3R3 in 200 EOC tissues. **d** RNA ISH assay of HOXD-AS1, miR-186-5p, PIK3R3 in HOXD-AS1 high expression ovarian cancer sample and HOXD-AS1 low expression ovarian cancer sample**. e**, **f** Kaplan Meier survival curve showing significantly poorer progression-free (**e**) and overall (**f**) survival in patients who showed high HOXD-AS1, low miR-186-5p and high PIK3R3 compared to patients who showed the reciprocal expression of these three factors. **g** Diagram summarizing the mechanism of action of HOXD-AS1/miR-186-5p/PIK3R3 on EMT in EOC cells

We proceeded to evaluate the clinical relevance of the in vitro HOXD-AS1/miR-186-5p/PIK3R3 pathway in 200 FFPE EOC samples. We observed significant inverse correlations between HOXD-AS1/miR-186-5-p (Fig. 6a) and between miR-186-5p/PIK3R3 (Fig. 6b) and significant positive correlation between HOXD-AS1 and PIK3R3 (Fig. 6c) using RT-qPCR quantitation. Additional in situ hybridization (ISH) data were shown to be consistent with RT-qPCR data (Fig. 6d). Furthermore, patients with high HOXD-AS1 (above the median value = 0.000565), low miR-186-5p (below the median value = 0.00000413), and high PIK3R3 (above the median value = 0.001908) expression showed significantly poorer PFS and OS compared to patients with low HOXD-AS1, high miR-186-5p, and low PIK3R3 (Fig. 6e and f). As summarized in Fig. 6g, in normal ovary cells, banlanced HOXD-AS1and miR-186-5p expression keeps EMT in check by regulating PIK3R3. In EOC cells, significantly elevated HOXD-AS1 level suppresses cellular miR-186-5p leading to increased PIK3R3 level which in turn leads to EMT.

## Discussion

Tumor metastasis is a major factor contributing to the poor prognosis of EOC patients [21, 22]. EMT is a crucial cellular process associated with metastasis in tumor cells [23, 24]. Our group has previously demonstrated that platinum-based drugs could induce the activation of Rac1 and initiate EMT [25]. Understanding the crucial players in the EMT process in EOC will provide insights into the development of new therapeutic strategies to treat the disease.

LncRNAs are an class of non-coding RNAs which plays an important regulatory role in the biological processes of tumorigenesis, tumor cell proliferation, invasion, and migration [15]. In EOC, aberrant expression of lncRNA regulates target genes through different mechanisms affecting biological behaviour of tumor cells. For example, lncRNA ABHD11-AS1 through regulating RhoC can promote A2780 and OVCAR3 proliferation and inhibit apoptosis [26]; lncRNA PTAR through miR-101 -3p/ZEB1 pathway behave in ceRNA model promotes invasion, migration, and EMT in EOC [27]; lncRNA PANDAR through SFRS2/p53 phosphorylation involved in the acquired Cisplatin resistance [28]. lncRNA aberrant expression in EOC can affect tumor proliferation, migration, and drug sensitivity. It also plays an important regulatory role in cancer initiation and progression.

lncRNAs have been divided into 5 categories based on the location of the lncRNA gene in the genome: (1) sense lncRNA, (2) antisense lncRNA, (3) bidirectional lncRNA, (4) intronic lncRNA, and (5) intergenic lncRNA [29]. In this study, we systematically investigated lncRNAs deregulation in EOC tissues versus normal ovary tissues and identified HOXD-AS1 to be significantly up-regulated in EOC tumors. HOXD-AS1 is an antisense lncRNA located on chromosome 2q31.2. Lu et al. have previously shown that HOXD-AS1 is over-expressed in metastatic hepatocellular carcinoma and promotes hepatocellular carcinoma metastasis through miR19a/ARHGAP11A signaling pathway [30]. Furthermore HOXD-AS1 has been demonstrated to cooperate with miR-130a to regulate SOX4 and E2F8, and promote metastasis in hepatocellular carcinoma and glioma respectively [31, 32]. In this study, we demonstrated that HOXD-AS1 acting as a cellular sponge in a ceRNA mechanism inhibits miR-186-5p and resulted in the corresponding up-regulation of PIK3R3 in EOC cells. This novel HOXD-AS1/miR-186-5p/PIK3R3 pathway plays an important role in EMT in EOC cells. In addition, high HOXD-AS1 level is significantly associated with poorer PFS and OS of EOC patients (Figs. 2 & 6), and hence demonstrating the clinical relevance of this novel pathway.

MiRNA-186-5p can bind to HOXD-AS1 and affect the expression of downstream target genes in the form of ceRNA. Abnormal expression of miRNA-186-5p in various tumors plays a regulatory role in the process of EMT. For example, high expression of miR-186-5p in colorectal cancer cells can inhibit the proliferation, metastasis, and EMT process of tumor cells; Expression changes of miR-186-5p are closely related to the proliferation and invasion ability of non-small cell lung cancer [33]. Therefore, the study further focused on whether HOXD-AS1 can regulate the expression of miR-186-5p through a ceRNA sponge model. Data of the present study suggests that HOXD-AS1 can negatively regulate miR-186-5p.

PIK3R3 is a regulatory subunit of PI3K, whose differential expression can promote the proliferation, invasion, and migration process of NSCLC cells, cervical carcinoma cells, and pancreatic cancer cells. Our studies have suggested that PIK3R3 is the target gene regulated by HOXD-AS1/miRNA-186-5p in the mode of ceRNA. PIK3R3 was down-regulated in SKOV3 cells transfected with si-PIK3R3 accompanied with the down-regulation of Twist and Rac1, which suggests Twist and Rac1 may mediate changes in PIK3R3 expression and affecting EMT in tumor cells [34–36].

In this study, we used lncRNA, microRNA, and mRNA biochips as carriers to discover a number of differentially expressed genes in ovarian cancer tissues. We utilized GEO, KEGG, DIANA and other common tumor databases for bioinformatics analysis. The results suggest that HOXD-AS1, as a ceRNA, upregulates PIK3R3 expression by sponging miR-186-5p. The higher expression of HOXD-AS1 is associated with shorter PFS and OS of EOC patients. Collectively, lncRNA-HOXD-AS1 may function as a promising biomarker and therapeutic target for human EOC treatment.

While this paper is in the process of preparation, Wang et al. reported HOXD-AS1 could regulate cell migration and invasion through miR-608 and FZD4 in ovarian cancer [37]. Therefore, these data suggest that HOXD-AS1 may function as a master regulator of metastasis through its interaction with a number of miRNAs and their respective target genes. HOXD-AS1 functions as a cellular sponge to modulate the availability of specific miRNAs at the 3’UTR of the miRNA target genes in ceRNA theory [20].

## Conclusions

In summary, we profiled the transcriptome landscape to report significant differentially expressed mRNA, lncRNA and miRNAs in EOC tissues compared with normal ovary tissues. We delineated a novel HOXD-AS1/miR-186-5p/PIK3R3 molecular pathway that regulates cell migration, invasion, and EMT process in EOC. Suppressing HOXD-AS1 can be a possible strategy to improve the clinical outcome of EOC patients.

## Additional files

Additional file 1: **Table S1.** The sequences of primers used for RT-qPCR assays. (DOCX 12 kb)

Additional file 2: **Figure S1.** miR-186-5P inhibition promotes EOC cell migration, invasion and EMT. (PDF 9600 kb)

Additional file 3: **Figure S2.** The statistical graph of western-blot. (PDF 4240 kb)

## Abbreviations

3’UTR: 3’Untranslated Region
ARHGAP11A: Rho GTPase activating protein 11A
CA125: Carbohydrate antigen 125
ceRNA: Competing endogenous RNA
CI: Confidence interval
DAVID: The Database for Annotation, Visualization and Integrated Discovery
E2F8: E2F transcription factor 8
EMT: Epithelial-esenchymal transition
EOC: Epithelial ovarian cancer
FBS: Fetal bovine serum
FDR: False discovery rate
FFPE: Formalin-fixed paraffin-embedded
FIGO: International Federation of Gynecology and Obstetrics
FZD4: Frizzled 4
GAPDH: Glyceraldehyde-3-phosphate dehydrogenase
GEO: Gene Expression Omnibus
GO: Gene Ontology
HOXD-AS1: HOXD antisense growth-associated long non-coding RNA
HR: Hazard ratio
KEGG: Kyoto Encyclopedia of Genes and Genomes
lncRNA: Long non-coding RNAs
miRNA: microRNA
PANDAR: The promoter of CDKN1A antisense DNA damage activated RNA
PBS: Phosphate buffer saline
PIK3R3: A regulatory subunit of phosphatidylinositol 3-kinase (PI3K)
PTAR: Pro-transition associated RNA
PVT1: Plasmacytoma variant translocation 1
Rac1: Rac family small GTPase 1
RhoC: Ras homolog gene family member C
RT-qPCR: Reverse transcription quantitative PCR
SAM: Significance analysis of microarray
SD: Standard error
SDS-PAGE: Sodium dodecyl sulfate- polyacrylamide gel electrophoresis
SFRS2: Arginine/serine-rich 2
SOX4: A member of the SOX (SRY-related HMG-box) gene family
Twist: Twist family bHLH transcription factor 1
XIST: X-inactive specific transcript
ZEB1: Zinc finger E-box binding homeobox 1
