# Supplementary material for: HOXD-AS1 promotes the epithelial to mesenchymal transition of ovarian cancer cells by regulating miR-186-5p and PIK3R3
Source: J Exp Clin Cancer Res. 2019 Mar 1;38:110. doi: 10.1186/s13046-019-1103-5 (PMC6397490; doi:10.1186/s13046-019-1103-5)
Supplement: Supplementary file 1 — Table S1. The sequences of primers used for RT-qPCR assays. (DOCX 12 kb) [file 13046_2019_1103_MOESM1_ESM.docx]

| Gene | Primer Sequence |
| --- | --- |
| HOXD-AS1 | forward primer: 5′- TTGGCTCTTCCCTAATGTGTGG-3′ |
|  | reverse primer: 5′- CCAGGTCCAGCATGAAACAGA-3′ |
| PIK3R3 | forward primer: 5′- ATGTACAATACGGTGTGGAGTATG-3′ |
|  | reverse primer: 5′- GCTGGAGGATCCATTTCAAT-3′ |
| Twist | forward primer: 5′- GTCCGCAGTCTTACGAGGAG-3′ |
|  | reverse primer: 5′- CCAGCTTGAGGGTCTGAATC-3′ |
| Rac1 | forward primer: 5′- TTACGCCCCCTATCCTATCCTATCC-3′ |
|  | reverse primer: 5′-CGCACCTCAGGATACCACTT-3′ |
| GAPDH | forward primer: 5′- GGCTGAGAACGGGAAGCTTGTCAT-3′ |
|  | reverse primer: 5′- CAGCCTTCTCCATGGTGGTGAAGA-3′ |
