# Supplementary material for: HOXD-AS1 promotes the epithelial to mesenchymal transition of ovarian cancer cells by regulating miR-186-5p and PIK3R3
Source: J Exp Clin Cancer Res. 2019 Mar 1;38:110. doi: 10.1186/s13046-019-1103-5 (PMC6397490; doi:10.1186/s13046-019-1103-5)
Supplement: Supplementary file 2 — Figure S1. miR-186-5P inhibition promotes EOC cell migration, invasion and EMT. (PDF 9600 kb) [file 13046_2019_1103_MOESM2_ESM.pdf]

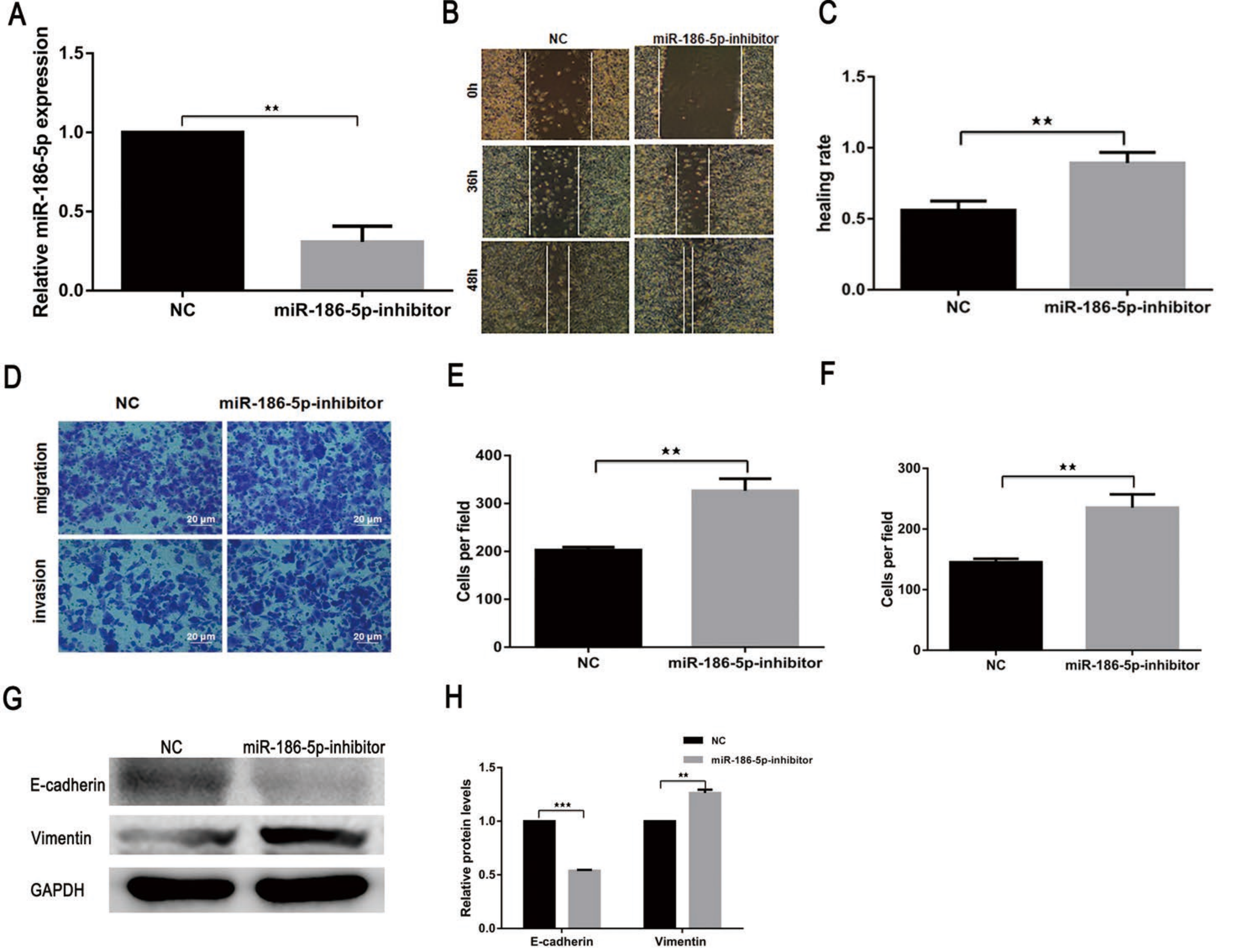

Figures S1 miR-186-5p inhibition promotes EOC cell migration, invasion and EMT.

A The expression levels of miR-186-5p in SKOV3 transfected with miR-186-5p inhibitor or NC by RT-qPCR.

B Wound-healing assays were used to examined the migration ability of SKOV3 after transfected with miR-186-5p inhibitor or NC.

C The statistical graph indicates the healing rate. The results are presented as the mean ± SD from three independent experiments.

DTranswell assays were performed to analysis the migration and invision ability of SKOV3 after transfected with miR-186-5p inhibitor or NC.

E/F The statistical graph indicates the number of cells averaged from 8 random high power fields after SKOV3 after transfected with miR-186-5p inhibitor or NC.

G/H The change of the protein levels of EMT-related genes after transfected with miR-186-5p inhibitor or NC.

Data were expressed as means ± SD from three independent experiments. ★$p<0.05$, ★★$p<0.01$, ★★★$p<0.001$.
