# Supplementary material for: HOXD-AS1 promotes the epithelial to mesenchymal transition of ovarian cancer cells by regulating miR-186-5p and PIK3R3
Source: J Exp Clin Cancer Res. 2019 Mar 1;38:110. doi: 10.1186/s13046-019-1103-5 (PMC6397490; doi:10.1186/s13046-019-1103-5)
Supplement: Supplementary file 3 — Figure S2. The statistical graph of western-blot. (PDF 4240 kb) [file 13046_2019_1103_MOESM3_ESM.pdf]

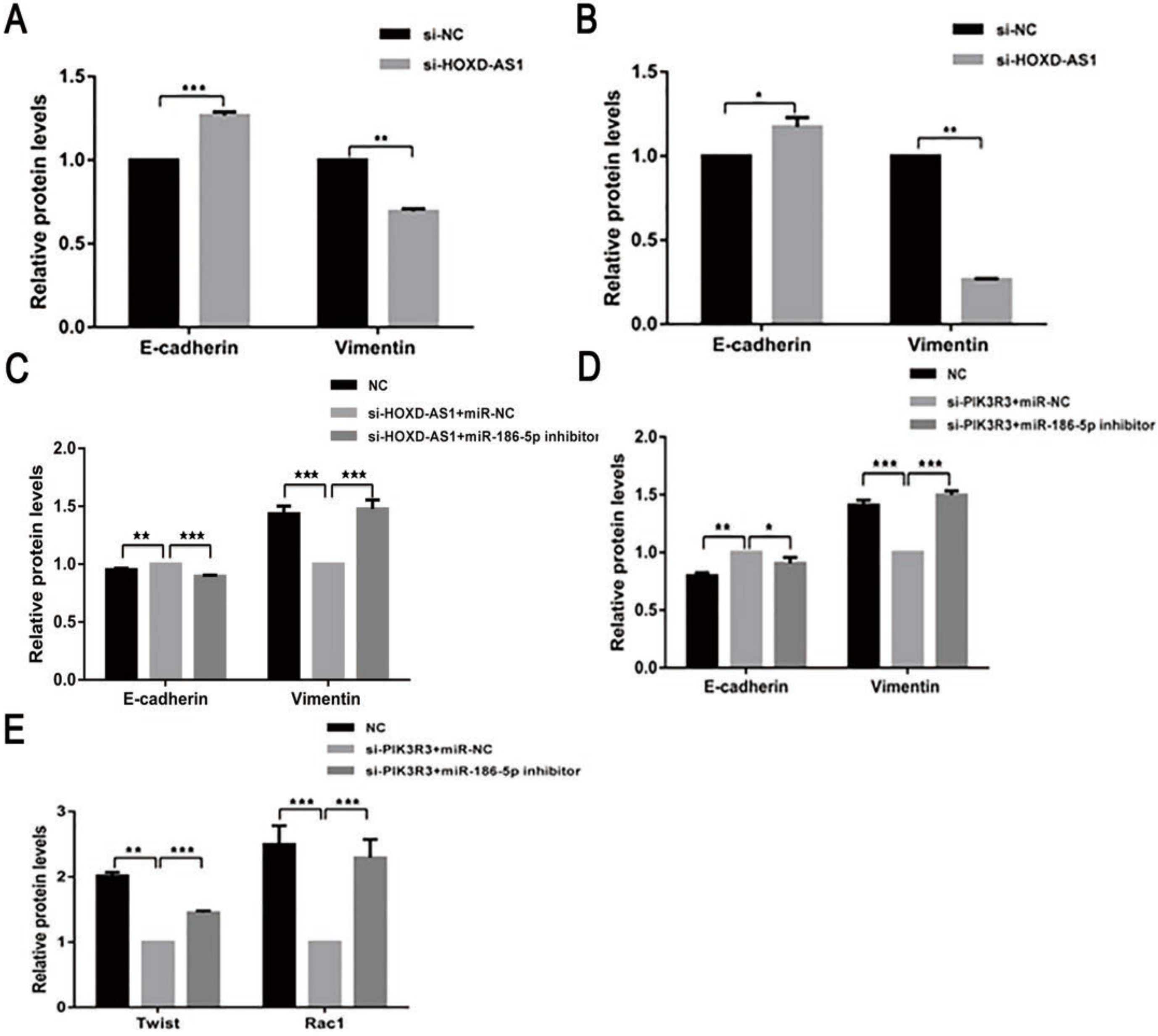

Figures S2 The statistical graph of western-blot.

A/B The statistical graph indicates the protein levels of EMT-related genes (E-cadherin, vimentin) in SKOV3 and A2780 cells transfected with HOXD-AS1 siRNAs or NC using western blot.

C The statistical graph indicates the protein levels of EMT-related genes (E-cadherin, vimentin) after SKOV3 cells transfected with si-NC, si-HOXD-AS1 with miR-NC or miR-186-5p inhibitor.

D The statistical graph indicates the protein levels of EMT-related genes (E-cadherin, vimentin) after SKOV3 cells transfected with si-NC, si-PIK3R3 with miR-NC or miR-186-5p inhibitor.

E The statistical graph indicates the protein levels of Twist and Rac1 after SKOV3 cells transfected with si-NC, si-PIK3R3 with miR-NC or miR-186-5p inhibitor.

Results were represented as protein intensity relative to GAPDH. Data were expressed as means ± SD from three independent experiments. ★$p<0.05$, ★★$p<0.01$, ★★★$p<0.001$.
